# Ceramide as a Promising Tool for Diagnosis and Treatment of Clinical Diseases: A Review of Recent Advances

**DOI:** 10.3390/metabo15030195

**Published:** 2025-03-11

**Authors:** Xueping Shen, Rui Feng, Rui Zhou, Zhaoyang Zhang, Kaiyong Liu, Sheng Wang

**Affiliations:** 1 School of Stomatology, Anhui Medical University, No. 81 Meishan Road, Hefei 230032, China; sxp@stu.ahmu.edu.cn; 2 Inflammation and Immune Mediated Diseases Laboratory of Anhui Province, Center for Scientific Research, Anhui Medical University, No. 81 Meishan Road, Hefei 230032, China; fr@ahmu.edu.cn (R.F.); 2022510018@ahmu.edu.cn (Z.Z.); 3 School of Pharmacy, Anhui Medical University, No. 81 Meishan Road, Hefei 230032, China; 2113051027@stu.ahmu.edu.cn; 4 School of Public Health, Anhui Medical University, No 81 Meishan Road, Hefei 230032, China; 5 Center for Big Data and Population Health, Institute of Health and Medicine, Hefei Comprehensive National Science Center, No 81 Meishan Road, Hefei 230032, China; 6 MOE Key Laboratory of Population Health Across Life Cycle, No. 81 Meishan Road, Hefei 230032, China; 7 Anhui Provincial Key Laboratory of Population Health and Aristogenics, No. 81 Meishan Road, Hefei 230032, China; 8 Center for Scientific Research, Anhui Medical University, No. 81 Meishan Road, Hefei 230032, China

**Keywords:** ceramides, cardiovascular disease, cancer, dermatosis, Alzheimer’s disease, obesity

## Abstract

**Background/Objectives:** Ceramide, a sphingolipid metabolite, has emerged as a key player in various physiological and pathological processes. Changes in ceramide levels are associated with the occurrence and development of various diseases, highlighting its potential as a biomarker of various clinical diseases. **Methods:** The biosynthesis and metabolism of ceramide are discussed, along with its functions in cell signaling, apoptosis, and inflammation. This study further examines the potential of ceramide as a biomarker for disease diagnosis and treatment. **Results:** This article highlights the involvement of ceramide in several diseases, including cardiovascular diseases, dermatosis, cancer, neurodegenerative disorders and metabolic syndromes. For each disease, the potential of ceramide as a biomarker for disease diagnosis and prognosis is explored, and the feasibility of therapeutic strategies targeting ceramide metabolism are reviewed. Additionally, the challenges and future directions in the field of ceramide research are addressed. **Conclusions:** This review article provides an overview of the recent advances in understanding the role of ceramide in clinical diseases and its potential as a diagnostic and therapeutic tool.

## 1. Introduction

### 1.1. Structure of Ceramide

**Figure 1:**
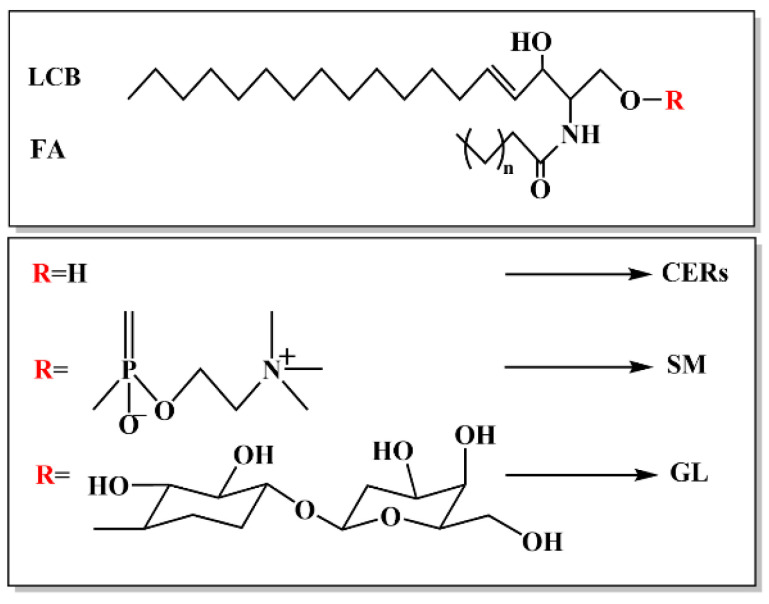
The structure of sphingolipids.

Ceramide is a bioactive sphingolipid with unique properties. Sphingolipids are classified into three subclasses: ceramide, sphingomyelin (SM), and glycosphingolipids (GLs), as shown in Figure 1 [1]. When the R is H, it forms ceramide. When the R is connected to choline phosphate or ethanolamine phosphate, it forms SM. By contrast, when this R is linked to glucose, galactose, or other sugars, it forms a GL. Typically, ceramide contains a fatty acid (FA) chain with varying even numbers of carbon atoms ranging from 2 to 28; the FA chain is connected to sphingosine (a long-chain base (LCB)) [2]. In an organism, ceramides exhibit diverse tissue distributions and biological functions due to differences in the carbon length and saturation of the FA chain, the degree of sphingosine hydroxylation, and the number and position of unsaturated bonds and hydroxyl groups. Thus, the unique bioactivity and biological functions of ceramides depend on their chain length and spatial distribution [3].

When describing the chemical structure, synthesis, and metabolic processes, we often use ceramide to refer to the whole ceramide classes, which better describe the shared characteristics of this sphingolipid. From a clinical perspective, however, we typically describe individual ceramides because different ceramides have different functions in diseases. By detecting the levels of specific ceramides, it is possible to more accurately diagnose certain diseases or assess risk and provide targeted treatment. For example, in the field of cardiovascular and cerebrovascular diseases, different types of ceramides (such as Cer(16:0), Cer(18:0), Cer(24: 0), and Cer(24:1)) can assess the risk of future cardiovascular and cerebrovascular adverse events in patients, enabling early warning and intervention. In dermatology, selecting products containing specific ceramides for treatment based on the type and severity of dermatosis can achieve better results.

### 1.2. Biosynthesis and Metabolism of Ceramide

**Figure 2:**
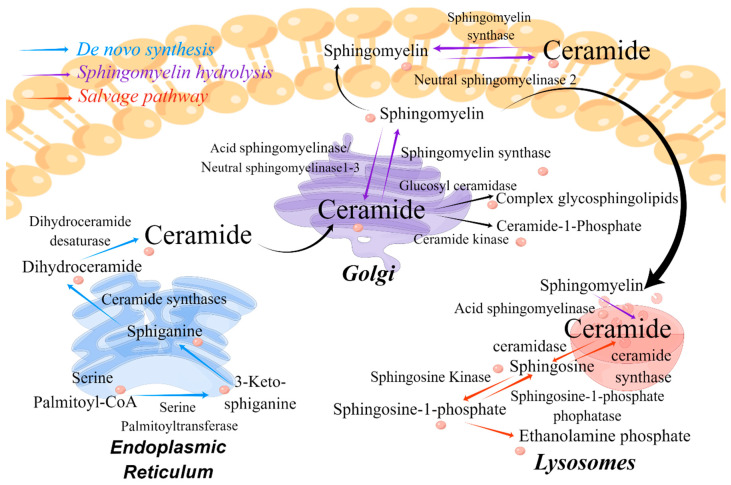
Synthesis and metabolism of ceramide in vivo.

Ceramide is produced through three typical pathways: de novo synthesis, salvage synthesis, and the SM hydrolysis pathway, as shown in Figure 2.

The de novo synthesis pathway is the main biosynthetic route [4]. This pathway occurs in the endoplasmic reticulum, and it is responsible for basal-level ceramide production in vivo over a longer time frame. Ceramide is synthesized from palmitoyl coenzyme A and serine in this pathway, which is catalyzed by serine palmitoyltransferase (SPT). The resulting product 3-keto-sphingosine is catalytically reduced to sphinganine by 3-keto-sphingosine reductase [5]. Because of the varying Acyl-CoA selectivity of six ceramide synthases (CerS1–6) in mammals, CerSs catalyze the acetylation of sphinganine to form dihydroceramides with varying chain lengths, ranging from medium- (C12–14), long- (C16–20), and very long- (C22–26) to ultra-long-chain FAs (>C26), and varying tissue distributions [4]. Subsequently, dihydroceramide is catalyzed by dihydroceramide desaturase to form ceramide, which is then translocated to the Golgi apparatus.

The salvage synthesis pathway is a rapid route for the production of ceramides. Sphingolipids are hydrolyzed by sphingomyelinase to form ceramide and choline phosphate. Ceramide can also be converted back into SM by the SM synthase through the addition of choline phosphate [5].

The SM hydrolysis pathway occurs in the plasma membrane, lysosomes, Golgi apparatus, and mitochondria. SM, an abundant sphingolipid in the plasma membrane, is degraded into ceramide and phosphorylcholine. On the basis of the pH environment, sphingomyelinases are classified into three types: acidic, neutral, and alkaline sphingomyelinases; ceramide synthesis requires at least acidic and neutral sphingomyelinases [5]. Under the influence of cytokines and chemotherapeutic drugs, neutral sphingomyelinase in cells is rapidly activated to produce ceramide in a short period, making it the central responder to stress-induced cell death. By contrast, acidic sphingomyelinase mediates radiation-induced apoptosis through the intracellular production of ceramide. In addition, sphingomyelin synthase (SMS) is a crucial enzyme for the catabolism of ceramide in the SM hydrolysis pathway, and the subcellular localization of its two subspecies, SMS 1 and SMS 2, differ [4].

### 1.3. Functions of Ceramide in Cell Signaling and Apoptosis

**Figure 3:**
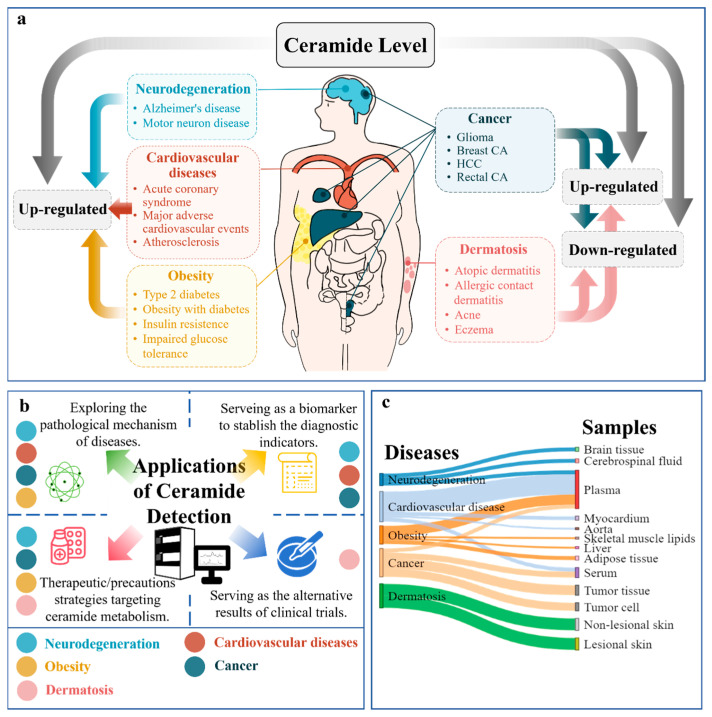
(**a**) Relationships between ceramide and clinical diseases; (**b**) Applications of ceramide detection; (**c**) Ceramide samples of the diseases.

Ceramide is a crucial component of sphingolipid metabolism, acting as a second messenger in cell signaling pathways and constituting an essential biochemical molecule. It can activate various kinases and transcription factors, leading to the regulation of cell growth, proliferation, differentiation, and apoptosis [6,7]. Ceramide-induced apoptosis is mediated through multiple mechanisms, including the activation of caspases, mitochondrial dysfunction [8], and endoplasmic reticulum stress [9]. Moreover, together with their upstream and downstream metabolites, ceramides contribute to the pathological processes of cancer, neurodegeneration, dermatosis, and cardiovascular disease (CVD), as shown in Figure 3a.

Given that the current studies typically emphasize the relationship between ceramide and one disease, this review provides an overall summary about various diseases, including cardiovascular diseases, dermatosis, cancer, neurodegenerative disorders, and metabolic syndromes. This review mainly focuses on the biomarker role of ceramides in diagnosis and prognosis, while all the aforementioned diseases have specific focuses, respectively. Additionally, the challenges and future directions in the field of ceramide research are addressed.

## 2. Application of Ceramide Measurement

Over the past 30 years, a growing body of evidence has revealed the regulatory role of the lipid ceramide in basic and clinical medicine due to its pivotal bioactivity [10]. Changes in ceramide levels are associated with the occurrence and development of various diseases, highlighting its potential as a biomarker of various clinical diseases. It is acknowledged that biomarkers refer to biological characteristics that can be objectively measured and evaluated in biological processes. Their main functions include disease diagnosis, risk assessment, treatment response monitoring, prognosis evaluation, etc. For example, an increased ceramide level in the blood is intensely related to CVD [11], and abnormally elevated plasma ceramide are associated with AD [12]. Furthermore, developing ceramide-targeting therapeutic strategies has attracted widespread research attention to tumor-targeting drugs focusing on regulating ceramide [13] and moisturizers containing ceramides [14] are convincing evidence. Moreover, choosing ceramide as the alternative results of clinical trials and exploring the mechanisms of diseases may be beneficial for further research, as shown in Figure 3b [15].

Ceramide levels can be measured in various biological samples such as blood, urine, and tissues, as shown in Figure 3c. Among the many analytical methods currently available, mass spectrometry is both highly specific and sensitive, and it has become an internationally recognized quantitative analytical technique for clinical study. Liquid chromatography–mass spectrometry (LC–MS/MS) is a specialized technique for the identification of compounds through the preparation, separation, and detection of gas-phase ions, and this method is widely applied for the detection of various ceramides in diverse fields [16]. While most of the ceramides described below have been detected through LC–MS/MS, there are other methods like mass spectrometry, quantitative tandem mass spectrometry (QTMS/MS), high-performance liquid chromatography–mass spectrometry (HPLC-MS/MS), ultra-high-performance liquid chromatography–tandem mass spectrometry (UHPLC-MS/MS) are typical for ceramide detection. In short, QTMS/MS mainly emphasizes the quadrupole tandem part in mass spectrometry technology without explicitly mentioning separation techniques. LC-ESI(APCI)-MS/MS is a type of liquid chromatography-tandem mass spectrometry technique that combines two different ionization sources: Electrospray Ionization (ESI) and Atmospheric Pressure Chemical Ionization (APCI). LC-ESI (APCI) MS/MS are based on liquid chromatography and tandem mass spectrometry techniques, which can help accurately identify and quantitatively analyze target molecules in samples, with higher separation efficiency and analysis speed. Our research group specifically developed a liquid–liquid extraction method last year, enabling the simultaneous detection of 35 ceramides in plasma samples, including Cer(18:1/2:0), Cer(18:0/2:0), Cer(18:1/4:0), Cer(18:0/4:0), Cer(18:1/6:0), Cer(18:0/6:0), Cer(18:1/8:0), Cer(18:0/8:0), Cer(18:1/10:0), Cer(18:1/12:0), Cer(18:0/12:0), Cer(18:1/14:0), Cer(18:0/14:0), Cer(18:1/16:0), Cer(18:0/16:0), Cer(18:1/18:1), Cer(18:1/18:0), Cer(18:0/18:1), Cer(18:0/18:0), Cer(18:1/20:0), Cer(18:0/20:0), Cer(18:1/22:0), Cer(18:0/22:0), Cer(18:1/24:1), Cer(18:1/24:0), Cer(18:0/24:1), Cer(18:0/24:0), Cer(12:0 (2R-OH)), Cer(16:0 (2R-OH)), Cer(18:0 (2R-OH)), Cer(18:1 (2R-OH)), Cer(20:0 (2R-OH)), Cer(22:0 (2R-OH)), Cer(24:0 (2R-OH)), Cer(16:0 (2R-OH)—note: this may be a repetition or error, as Cer(16:0 (2R-OH)) is already listed), Cer(d18:1-d7/6:0), Cer(d18:1-d7/16:0), and Cer(d18:1-d7/24:0). Each of these ceramides has a corresponding reference compound for comparison. Here, we merely provide several alternative methods for ceramide detection in Table 1.

### 2.1. Ceramide and CVD: Ceramide Scoring Criteria Have Been Established

CVD is a prevalent chronic disease that poses a substantial threat to human health, with morbidity and mortality rates increasing annually. Under normal conditions, free FAs in the body are converted into ATP through β-oxidation. Excess free FAs that exceed the capacity for β-oxidation are connected to glycerol backbones, producing inert triglycerides. These inert lipids are subsequently stored in intracellular fat droplets without causing any damage. However, when the capacities for these mechanisms are chronically saturated, detrimental, physiologically active FAs accumulate. When excess lipids accumulate in nonadipose tissues, such as blood vessels and the heart, they impair cardiac and vascular function, leading to pathological processes such as atherosclerosis. Among these deposited lipids, sphingolipids, particularly ceramides, exert the most damaging effects on the cardiovascular system [8]. Typically, ceramides play a structural role in cell membranes. Basal levels of ceramides provide signals indicating the abundance of intracellular free FAs and initiating a response to the lipid burden on the effect of the physiological and nutritional stress [8]. Ceramides are metabolized into compounds (e.g., sphingosine-1-phosphate [S1P]) that can alleviate the lipid burden [11]. However, long-term ceramide accumulation promotes the activation of oxidative stress pathways in endothelial cells. High ceramide levels are associated with vascular endothelial damage, cardiometabolic disease, and early adverse cardiovascular events [11].

Accurately predicting the risk of CVD is essential for its prevention. Although angiography is commonly used in clinical practice for the precise diagnosis of CVD, it is invasive and expensive. Electrocardiography and rating scales can also be used to assess CVD risk, but their implementation requires practitioners with extensive theoretical knowledge and practical experience [26]. Furthermore, widely used tools such as the Framingham Comprehensive Cardiovascular Risk Score (FRS) and the SCORE scale have limitations [27]. For example, the SCORE scale considers only one marker, namely cholesterol, and clinical studies have demonstrated that cholesterol levels do not effectively reflect a patient’s risk of CVD. Moreover, total cholesterol and low-density lipoprotein cholesterol (LDL-C) fail to predict future cardiovascular events in high-risk patients [27].

In recent years, an increasing number of studies have reported an association of ceramides with various cardiovascular risk factors. The detection of specific ceramides can efficiently and accurately predict cardiovascular events; thus, ceramides can be applied as biomarkers of cardiovascular health [8,28].

Currently, the Ceramide Risk Scoring System (CERT1 score), Ceramide-Phosphatidylcholine Risk Scoring System (CERT2 score), and High-Sensitive Troponin T Scoring System (CERT2-TnT score) are CVD scoring criteria that include ceramide indicators [27]. Table 2 lists the indicators and calculations used in these three ceramides risk scoring systems [29]. In the CERT1 scoring system, three ceramide concentrations and three ceramide ratios are selected as scoring variables. The scores range from 0 to 12, which are determined according to quartiles: ceramide concentrations (or ratios) in quartile Q4 are assigned a score of 2, those in Q3 are assigned a score of 1, and those in Q2 and Q1 are assigned a score of 0. Patients can then be classified into four categories of mortality risk (low, intermediate, moderate, and high) corresponding to the scores of 0–2, 3–6, 7–9, and 10–12, respectively [29]. According to this scale, the risk of cardiovascular mortality is 4.2 to 6 times higher in high-risk patients than in low-risk patients. According to the CERT2 score, high-risk individuals have a 3.5 to 4 times higher risk of cardiovascular death than low-risk individuals. According to the CERT2-TnT score, the risk of cardiovascular death is >10 times higher in high-risk individuals than in low-risk individuals. In addition, a study indicated that CERT1 scores, serum levels of soluble growth-stimulating expressed gene 2, N-terminal brain natriuretic peptide precursor, and frailty substantially affect the occurrence of adverse cardiovascular events in older patients with chronic heart failure. These factors individually have high prognostic predictive value, and their combined predictive value is even higher [30]. Liu et al. reported that the high levels of Cer(16∶0), Cer(18∶0), and Cer(24∶1) were associated with the occurrence of atherosclerosis. High plasma ceramide level is an independent risk factor for atherosclerosis [16]. Ge Junbo et al. prospectively investigated the potential diagnostic value of ceramide for acute coronary syndrome (ACS) among Chinese patients with chest pain. They found that after adjusting for traditional risk factors and markers of myocardial injury (e.g., high-sensitivity troponin), the ratios of Cer(d18:1/24:1(15Z)) to Cer(d18:1/24:0), Cer(d18:1/14:0), and Cer(d18:1/22:0) were independent predictors of ACS diagnosis in patients with chest pain [31]. Cheng et al. reported that Cer(d18:1/16:0) was significantly associated with the core necrotic tissue fraction and lipid core burden in coronary atherosclerosis and that this ceramide could more accurately predict clinical outcomes in patients 1 year after coronary angiography [32]. The SIC (sphingolipid-inclusive CAD risk) score developed by Poss et al. is more effective than traditional clinical biomarkers of CVD. This score evaluates ceramide species, such as Cer(d18:1/24:0), Cer(d18:1/18:0), and Cer(d18:1/18:0)/Cer(d18:1/22:0) ratios, and SM species, such as SM(d18:1/24:0) and the SM(d18:0/24:1)/sphingolipid(d18:1/18:0) ratio [33].

The measurement of ceramide levels is likely to become an increasingly applied technique for the clinical diagnosis and treatment of CVD with advances and the clinical application of mass spectrometry [27,28]. However, further investigation into several aspects is required for improving the assessment criteria for ceramide as markers of CVD. The first aspect is how to enhance predictive value by combining various assessment criteria. A study demonstrated that combining the CET2 and SCORE scores, with the weighting of the CET2 score at 1.5 and the SCORE at 1, to create the ESC-CERT2 scale resulted in higher predictive value than either score [34]. The second aspect is developing appropriate evaluation criteria for different high-risk patient groups. Specific CVD risk prediction models have been developed for various populations, including individuals with cardiovascular risk factors (diabetes and obesity) and pregnant individuals. However, whether ceramide levels differ among these populations and whether they can be included in evaluation indices remains to be determined. Many researchers have combined medical data with artificial intelligence and machine learning models to establish novel digital medical technologies [26,35]. Similarly, risk factor scales and diagnostic models that incorporate ceramide levels should be established for predicting disease risk in healthy populations, hospital readmission rates, and mortality rates in susceptible groups. Exploring metabolic changes and patterns in ceramide in different stages of a disease can provide a basis for disease classification or staging, aligning with the requirement of precise and individualized diagnosis and treatment [27]. Since the 20th century, LDL-C levels have been used as key biological indicators for predicting CVD, and LDL-C- and cholesterol-lowering drugs, such as statins and fibrates, are effective as mainstream treatments [8]. Although plasma ceramide levels are effective predictors of CVD, the currently available lipid-modifying drugs targeting sphingolipids (particularly ceramides) have not yet been extensively studied. Thus, ceramide as a target of drug therapy for CVD must be urgently investigated. Further research is required to determine the effect of the reduction of plasma ceramide levels on the efficacy of current first-line medications [8].

### 2.2. Ceramide and Cancer: Insights into Application of Sphingolipid-Based Drugs

Tumors arise when cells proliferate abnormally and malfunction due to DNA mutations caused by long-term exposure to internal or external carcinogenic factors. This phenomenon leads to the loss of control over cell growth and division. By participating in numerous cellular activities, sphingolipids play crucial roles in regulating the physical properties of eukaryotic cell membranes and function as signaling molecules in response to physiological signals and stresses. Ceramide, a biologically active lipid, plays a crucial role in the membranes of organelles and the plasma membrane, and it is a key molecule in sphingolipid metabolism. Changes in ceramide levels at the plasma membrane generate signals for regulating cellular functions, including inhibiting cell proliferation and promoting cell differentiation and apoptosis [36]. In cancer cells, ceramides also regulate molecules involved in anti-inflammatory responses, apoptosis, programmed necrosis, autophagy, and cytokinesis [7,37]. The dysregulation of ceramide metabolism is frequently observed in various cancers. Table 3 presents the ceramide levels observed in patients with cancer.

Ceramide levels in plasma or tumor tissues are valuable as effective clinical biomarkers and for assessing tumor progression and prognosis. However, additional large-scale studies and prospective validation are still needed. Metabolomics is likely to become a standard technique in translational cancer research in the future [43]. Studies have reported that lipid metabolomics combined with bioinformatics can be used to identify the putative biomarkers of specific metabolic pathways related to tumor diagnosis, classification, and prognosis. Statistical methods and pathway enrichment analysis [43] can be employed for identifying metabolic pathways in different stages of cancer, revealing dynamic changes in lipids (including ceramides) in colorectal cancer [43], hepatocellular carcinoma [44], gastric cancer [45], and epithelial ovarian cancer [46], thus aiding in the establishment of a lipid metabolic profile.

**Figure 4:**
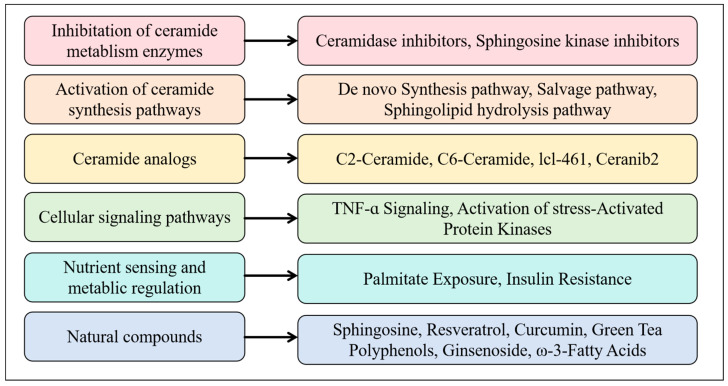
Approaches for modulating ceramide levels in cancer cells.

Ceramides have attracted widespread research attention as potential tumor suppressor drugs. Currently, tumor-targeting drugs focus on regulating ceramide levels in cancer cells [7]. Figure 4 presents these approaches.

Since the discovery of Cer6 as a proapoptotic lipid by Obeid et al. [13], increased attention has been paid to ceramide-based cancer therapies. Multiple stress stimuli, including ionizing radiation, chemotherapeutic agents, and TNF-α, can increase ceramide levels by activating the de novo synthesis and SM hydrolysis pathways of ceramides [47]. Thus, in addition to serving as a biomarker of cancers, ceramides should be assayed for studying tumor therapeutic mechanisms.

Ceramide- and SM-metabolizing enzyme inhibitors (e.g., salfingol and anti-sphingomyelinase antibody) increase the sensitivity of head and neck squamous cell carcinoma to radiotherapy and chemotherapy by elevating ceramide levels in tumors; thus, these inhibitors serve as sensitizers for radiotherapy and chemotherapy [48]. However, ceramide production in tumors and tumor vasculature is low at a radiotherapy dose of 2 Gy [49]. Additional studies on the interaction between the X-ray irradiation dose and ceramide production are required [48].

A recent study revealed that ultrasound-stimulated microbubbles can enhance ceramide-based radiation effects by mechanically perturbing cell membranes. This technique led to the accumulation of ceramides in endothelial cells as well as in leukemia, breast, prostate, and fibrosarcoma cells and promoted ceramide production in deep tumor tissues [48].

Combination therapy has become a major trend in tumor treatment strategies. In this treatment approach, ceramide modulators are used in conjunction with chemotherapeutic agents, immunotherapy, apoptosis inducers, and antiangiogenic drugs. These combinations exert synergistic antitumor effects, enhancing cytotoxicity and improving treatment sensitivity [7]. However, some ceramide metabolites, such as glucosylceramide, S1P, and ceramide-1-phosphate, can promote cell proliferation and chemoresistance. To tackle this, modulators of enzymes, such as SPT, glucosylceramide synthase, SMS, and ceramidase, should be included in combination therapy. In recent years, ceramide-metabolizing enzymes have become new targets for cancer therapy [50].

Because systemic administration may lead to off-target effects and potential in vivo toxicity, ceramide-targeted delivery to cancer cells should minimize these effects on normal cells. Currently, c6-ceramide-based stealth liposome formulations, such as ceramide nanoliposomes (CNLs), and receptor-targeted liposome formulations, such as transferrin-coupled ceramide liposomes, have been investigated for their effective targeted delivery. Among these formulations, CNLs are potential chemotherapeutic agents that can induce necroptosis by activating mixed lineage kinase domain-like protein (MLKL). The in vivo efficacy of CNLs for various types of cancers, including gastric cancer, hepatocellular carcinoma, ovarian cancer, and leukemia, has been demonstrated. However, the underlying molecular mechanisms remain poorly understood, and these mechanisms should be further explored through ceramide assays [13].

Drug resistance is among the main obstacles to tumor treatment, which implies that tumor cells can develop mechanisms to evade ceramide-induced apoptosis. Methods to prevent or overcome drug resistance are crucial for the long-term efficacy of ceramide-based therapies. Rigorous pharmacokinetic, pharmacodynamic, and potential off-target effect studies should be conducted for developing these methods [7]. By determining the intracellular levels of ceramides and ceramide metabolites as well as the related enzyme activities after cancer treatment, researchers can elucidate the drug mechanisms and pathways for the clinical application of sphingolipid-based drugs [38,51].

### 2.3. Ceramide and Dermatosis: A Biomarker and Key Component in Skin Permeability Barrier

The skin, the largest organ, plays vital roles in terms of its protective barrier and sensory perception functions, and the skin immune system. Lipids constitute 5%–15% of the stratum corneum, functioning as the “mortar” that immobilizes the keratinocyte-formed “bricks” [52]. Lipid-bonded squamous cells contribute to the strong barrier function of the skin [53]. Ceramide, a key physiological lipid, is synthesized and secreted by lamellar bodies (Odland vesicles) and constitutes 40%–50% of the weight of stratum corneum lipids (with cholesterol accounting for 25% and free FAs 15%) [54]. As the most abundant lipid component in the stratum corneum, the appropriate amount and type of ceramides should be maintained for ensuring an effective skin barrier [55].

**Figure 5:**
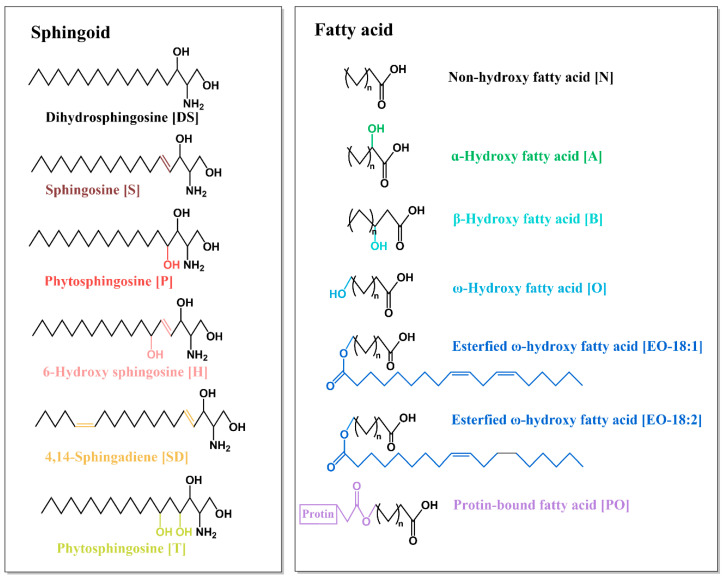
Structures and nomenclature of ceramide in human SC. Note: [DS]: dihydrosphingosine; [S]: sphingosine; [P]: phytosphingosine; 6-hydroxy [H]: sphingosine; [SD]: 4,14-Sphingadiene; [N]; non-hydroxy fatty acid; [A]; ɑ-Hydroxy fatty acid; [B]; β-Hydroxy fatty acid; [O]: ω-Hydroxy fatty acid; [EO]: esterfied ω-hydroxy fatty acid; [PO]: protein-bound fatty acid.

Currently, two nomenclature systems are commonly used for ceramides in dermatology. The first method is based on the order of chromatographic migration, naming ceramides 1–8 in sequence, with ceramides 9–11 named according to the chronological order of their discovery. In the second method, ceramides are classified based on their molecular structure, and each ceramide is designated using abbreviations that represent the structure of the LCB and FA, as shown in Figure 5. According to this method, ceramides are divided into 25 classes, with letters representing the acyl chain: [N] (nonhydroxy), [A] (α-hydroxy), [B] (β-hydroxy), [O] (ω-hydroxy), [EO] (esterified omega-hydroxy), and [PO] (esterified omega-hydroxy). The final letter refers to sphingosine moieties, such as [S] (sphingosine), [DS] (dihydrosphingosine), [P] (phytosphingosine), [H] (6-hydroxy-sphingosine), and [SD] (4,14-sphingosine) [56], as listed in Table 4 [55]. In mammals, the most abundant ceramides are composed of sphingosine and nonhydroxylated FAs and are known as CER[NS]. Both CER[NS] and CER[NDS] are present in almost all mammalian tissues, whereas other ceramides are found only in specific tissues [55]. Previously, the human skin stratum corneum was believed to contain only 16 ceramide subtypes, excluding those containing [SD], [B], and [PO], with 12 being free-extractable subtypes and 4 being ω-hydroxylated sphingosines (i.e., CER[ODS], CER[OS], CER[OP], and CER[OH]) bound to the cellular cuticle, making them difficult to extract and measure. In addition, the low content of CER[EODS] made it challenging to measure. Thus, earlier studies have typically reported data on only 11 ceramide subtypes [56]. Ceramides containing β-hydroxy FAs have been found in mouse epidermis but not in human skin [57]. Furthermore, some EO ceramides are converted into protein-bound ω-hydroxy ceramides (P-O ceramides), which are linked to crosslinked proteins in the keratinized envelope. Damage to EO or P-O ceramides can lead to ichthyosis [55]. Recently, the number of ceramide subclasses in the stratum corneum of the human skin has expanded to 21, including SD ceramides, which contain a 4,14-sphingodienyl base and were first identified in the human skin [55]. With advancements in analytical techniques and more sensitive methods, additional ceramide bases can be discovered, such as the recently identified 1-O-acyl ceramide [1-O-E(EO)Cer] in EpiSkin, a human reconstructed epidermal model [58]. Characterizing the fine structure of ceramide biomarkers is essential for tracking and studying metabolic pathways, and developing new assays to study lipid biosynthesis and lipids in the stratum corneum can explain the impaired barrier function of the skin caused by disruptions in these pathways [58].

Altered ceramide profiles are closely linked to the development of skin diseases associated with impaired barrier function, such as atopic dermatitis, psoriasis [36], acne, and autosomal recessive congenital ichthyosis. Moreover, abnormal ceramide metabolism is implicated in rare skin disorders, including Dorfman–Chanarin syndrome and Sjögren–Larsson syndrome [56]. Table 5 displays the levels of skin or plasma ceramides in various dermatological conditions, highlighting the variations in ceramides and other lipids between the test group and the control group.

In dry skin relative to normal skin, the ceramide level is significantly lower, or the type of ceramide may change [1]. However, specific ceramide levels could have a discrepancy among different studies resulting from the characteristics of the populations, including race, age, and severity of diseases. For example, though one study has shown that children with AD family history the amounts of specific CER subclasses, such as CERs with sphingosine N-acylated with α-hydroxy FA (AS-CERs) and NS-CERs, were elevated in Chinese children, another study reported no differences in the amounts of AS-CERs and NS-CERs between the AD and control groups, while the levels of total CERs and EOS-CERs were decreased in AD patients [64].

For maintaining skin barrier integrity, ceramides have become the preferred option over corticosteroids [68]. The topical application of moisturizers containing ceramides, particularly ceramide 3, can alleviate dry skin symptoms and restore skin barrier function. Although ceramides are beneficial for the clinical management of dermatological problems, there is a lack of research on the optimal lipid ratios for various conditions. Ceramide levels in the stratum corneum can serve as an experimental index for evaluating skin permeability barrier function [66], guiding research on pathophysiology, treatment plans, and efficacy assessments [15]. Although numerous studies have demonstrated the role of ceramides in the skin permeability barrier, additional research is still required to determine the acceptable range of variation in the ceramide composition while maintaining barrier function. In addition, although the ceramide profile of other mammals (such as mice, guinea pigs, dogs, and pigs) is quite different from normal humans, these animals still possess an effective permeability barrier in the healthy state, highlighting the need for more research in this area [36].

### 2.4. Ceramide and Alzheimer’s Disease: Exploring Relationships Between Lipid Dysregulation and Neurodegenerative Diseases’ Mechanism

Alzheimer’s disease (AD) is one of the most common neurodegenerative disorders, affecting millions of people worldwide, with both prevalence and incidence increasing with age. AD is mainly characterized by cognitive decline and memory loss; thus, it is the leading cause of dementia. Although the exact pathogenesis of AD remains unclear, its pathological features, including the presence of neurofibrillary tangles formed by hyperphosphorylated Tau proteins and extracellular amyloid peptide aggregates, are well established. Based on these key hallmarks and symptoms, AD can be categorized into three stages: preclinical AD, mild cognitive impairment (MCI) due to AD, and dementia, which is shown in Table 6 [69].

AD is primarily diagnosed through cognitive tests, neuroimaging, and biomarker screening. Ideally, a diagnosis should be made before the onset of symptoms by using minimally invasive and low-cost techniques, such as cerebrospinal fluid (CSF) biomarkers combined with amyloid PET imaging. An ideal biomarker should meet several criteria, including (i) high sensitivity and specificity to accurately differentiate AD from other dementias as well as high reliability; (ii) a positive predictive value; (iii) involvement in the pathology of AD; (iv) the abilities to enable early detection, provide diagnostic and prognostic information, and optimize therapeutic strategies for patients; and (v) availability through noninvasive and cost-effective methods [69].

**Figure 6:**
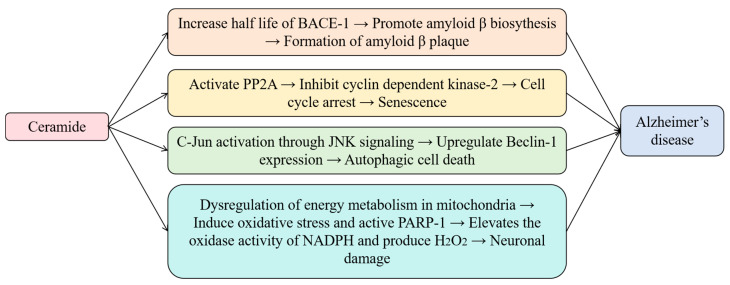
Mechanistic role of ceramide in the pathogenesis of AD.

Metabolic dysregulation is associated with the development and progression of early neurodegenerative diseases, including AD [70]. Lipids are highly abundant in the brain, constituting >50% of its dry weight [71], and play a crucial role in both physiological and pathological processes. Alterations in lipid metabolism are considered a key factor in AD pathogenesis [72]. Sphingolipids, in particular, are enriched in the central nervous system (CNS) [73], and they were first identified in the brain tissue. In 1884, the German physician Johann Ludwig Wilhelm Thudichum isolated a previously unknown lipid species from brain tissue. Due to its unique amphiphilic properties and unknown biological roles, he named the obtained species as sphingolipids after the mythical Sphinx of Greek legend [74]. Studies have demonstrated the abundant distribution of sphingolipids in the membranes of nerve cells and the myelin sheaths of nerve fibers [72], and sphingolipids also play a crucial role in AD pathogenesis. In the CNS, ceramide, a key bioactive lipid within the sphingolipid family, is synthesized in the endoplasmic reticulum of neurons and glial cells [73]. Ceramide levels both in cells and plasma significantly affect several pathological features of AD, including amyloid-beta (Aβ) plaques, mitochondrial dysfunction, cellular senescence, autophagy dysfunction, platelet activation, and endothelial dysfunction [73]. The mechanistic role of ceramide in the pathogenesis of AD shown in Figure 6 [69].

Current research suggests that the dysregulation of the ceramide synthesis pathway and the disruption of the role of ceramide in maintaining cellular homeostasis can ultimately contribute to the development of AD [72]. Elevated plasma ceramide levels are associated with cognitive decline in patients with AD [12,75], and plasma ceramides are potential biomarkers for predicting and diagnosing AD [76,77]. The alterations in ceramide and its metabolites as well as in other lipids in AD are shown in Table 7.

Numerous studies have demonstrated that the etiology and progression of various neurodegenerative and psychiatric disorders are linked to the dysregulation of lipid metabolism in the brain; disruptions in the sphingolipid metabolism significantly influence age-related neurodegenerative diseases [70,80,81]. For example, abnormalities in ceramide-related metabolites have been observed in both Huntington’s disease (HD) and schizophrenia (SCZ). In HD, elevated SM kinase levels have been found in brain tissue, whereas increased serum SMs have been detected in SCZ [82]. Alternations in ceramide levels in other neurodegenerative diseases are shown in Table 8.

In addition to altered ceramide levels in plasma and brain tissue, CSF samples may serve as valuable indicators of the pathology of brain disorders because CSF contains primary metabolites that have diffused from the brain. Lipids in the CSF and blood have been recognized as potential markers of diseases such as AD, Parkinson’s disease (PD), and SCZ [88]. Elevated ceramide levels in CSF have been observed in neurodegenerative disorders, including AD and amyotrophic lateral sclerosis [89].

In the brain, the dysregulation of lipid metabolism is associated with not only neurodegenerative diseases but also the pathology and progression of other neurological conditions. Ceramides play a role in the pathophysiological mechanisms of mood disorders. Historically, bipolar disorder (BD) was often misdiagnosed as major depressive disorder (MDD) due to overlapping symptoms and the lack of objective diagnostic tools. However, recent studies have identified plasma Cer(d18:0/24:1) as a strong blood biomarker for distinguishing between BD and MDD. When combined with patient-reported information to complement psychometric assessments, this biomarker significantly improves diagnostic accuracy, suggesting intrinsic differences in immunometabolism or signaling between BD and MDD that warrant further investigation [90]. Moreover, the elevated serum levels of the C16-ceramide are associated with an increased risk of depression in patients with cerebral hemorrhage; thus, C16-ceramide is a promising biomarker for diagnosing posthemorrhagic depression [88].

Research on the effects of altered sphingolipid metabolism on the development of neurodegenerative diseases suggests that modulating sphingolipid metabolism is a promising therapeutic approach for these diseases [91]. Drugs targeting the ceramide metabolic pathway, which are currently available in the Westpeptide database, include myoglobin derivatives, fingolimod, FTY720, and flucytosine hydrochloride [12]. Among these drugs, FTY720 mimics the biological activity of S1P, although its efficacy for AD treatment remains unclear. Through sphingolipid profiling, RT-PCR for gene expression, and ELISA for Aβ quantification, studies have demonstrated that FTY720 exerts therapeutic effects by inducing a shift in brain sphingolipids toward low-toxicity metabolites (e.g., Cer(d18:1/16:0) and Cer(d18:1/22:0)) and by reducing the Aβ burden and inflammation [92].

In summary, the detection of ceramide levels in plasma, brain tissue, or CSF has crucial implications for understanding the role of the dysregulation of lipid metabolism in brain diseases and for developing ceramide-targeted therapies.

Although ceramide assays have been widely performed in various neurodegenerative diseases and psychiatric studies, several key points warrant attention. First, ceramides with different acyl chain lengths and saturations have distinct physiological roles, but the specific role of individual ceramides in neurological disorders remains unclear [73]. Second, ceramide levels are not constant during disease processes; the same ceramide can exhibit opposite metabolic manifestations in different conditions (e.g., in a traumatic brain injury mouse model, Cer(22:0) decreased at 1 day but increased at 7 days) [87], rendering the dynamic detection of ceramide levels crucial for investigating disease progression. Additionally, variability in lipidomic metabolite levels across studies indicates the need for the standardization of each metabolite. When combined with artificial intelligence techniques and interdisciplinary collaborative research, such as incorporating sphingolipid data with machine learning-based statistical methods [93], lipidomics can enable the identification of the factors closely associated with disease, facilitating the treatment of clinically heterogeneous brain disorders [82]. Finally, due to the complexity of lipid metabolism, the consistency of sphingolipid level changes in the CSF and blood remains a topic of debate that requires further investigation. In small-scale or cross-sectional studies, only correlations between variables can be determined and not causality. Therefore, longitudinal studies and larger case studies should be performed to provide more comprehensive insights [85].

### 2.5. Ceramide and Metabolic Syndromes: Promote Better Understanding of Disease and Treatment Mechanism

Ceramide is involved in the development of metabolic syndromes such as insulin resistance, diabetes, and obesity. Obesity is characterized by the storage or ectopic deposition of excess energy ingested by the body in the form of fat, which is accompanied by metabolic symptoms such as elevated blood glucose levels and lipid metabolism disorders. The altered lipid metabolism caused by obesity is a major risk factor for chronic diseases such as metabolic syndrome, which has become an increasingly severe public health concern worldwide [3]. Obesity is also a major contributor to the development of insulin resistance (IR) and T2D. Furthermore, obesity is associated with adipocyte dysfunction, macrophage infiltration, and low-grade inflammation, all of which may contribute to the onset of IR. In the normal physiological state, the adipose tissue synthesizes and secretes various bioactive molecules such as adipokines and cytokines, which regulate lipid and glucose metabolism. In obesity, disruptions in adipokine and cytokine production lead to alterations in lipid and carbohydrate metabolism, potentially contributing to the onset of IR and the development of T2D. Obesity is also associated with the accumulation of lipids, particularly long-chain acyl coenzyme A, ceramides, and diacylglycerols. These lipids regulate intracellular enzyme activity, and their accumulation in adipocytes may be linked to the development of IR [94]. Although sphingolipids, specifically ceramides, are not the most abundant lipids in mammalian cells, the accumulation of ceramides in insulin-sensitive tissues and the islets of Langerhans during insulin toxicity is believed to play a crucial role in the onset and progression of metabolic disorders [95]. Ceramides may contribute to the development of these disorders through mechanisms such as inhibiting insulin signaling, promoting apoptosis, disrupting mitochondrial function, regulating inflammatory responses, and ultimately inducing IR and promoting the development of obesity and obesity-related diseases [3].

Numerous studies have identified significantly elevated ceramide levels in individuals with obesity and obesity-related diseases, shown in Table 9. Specifically, lipids can contribute to ceramide metabolism through four factors. First, due to the oversupply of saturated or unsaturated FAs, ceramides may be synthesized through the sphingolipid cycle or the increased activity of the salvage pathway in tissues. For example, among saturated FAs, palmitate is among the most prevalent FAs in circulation, and the chronic exposure of cells to palmitate can lead to the formation of Cer(d18:1/14:0), Cer(d18:1/16:0), and Cer(d18:1/24:0) through sphingosine acylation, thereby impairing cell function and mass [96]. Second, increased lipolytic activity in hypertrophied adipose tissue leads to increased levels of FFAs, which inhibit insulin action [97], and elevated plasma levels of FFAs promote the synthesis of ceramide through the de novo pathway by increasing the activity of ceramide-metabolizing enzymes [96]. For example, in patients with diabetes, the activity of nSMase (an enzyme involved in ceramide production) is increased, and aSMase is inhibited. In leptin-deficient, genetically obese (ob/ob) mice, obesity-associated hyperinsulinemia and increased TNFα resulted in the increased expression of the three main enzymes involved in ceramide production (nSMase, aSMase, and SPT) in adipose tissue. In the adipocytes of obese individuals, higher ceramide levels may result from the higher activity of enzymes involved in ceramide production (SPT, nSMase) [98]. In women with obesity, the increased activity of nCDases in adipose tissue serves as a compensatory mechanism that reduces ceramide accumulation in adipocytes [99]. Third, the elevated plasma levels of FFAs activate Toll-like receptors (TLRs) in macrophages, producing various cytokines that regulate ceramide synthesis [100]. Fourth, saturated FAs induce ceramide synthesis and inflammatory responses in a TLR4-dependent manner.

Ceramide assays can be vital for exploring both the mechanisms of disease progression and the effects of obesity treatments. Although the pathophysiology of T2D has been extensively studied, its molecular mechanisms remain incompletely understood. Lipidomics can provide a detailed assessment of biomolecular features through quantitative and qualitative analyses. By integrating genomic data, including lipidomics, metabolomics, and proteomics, researchers can explore the close relationship between ceramides, metabolites, and the metabolic environment. This approach facilitates further investigation into the metabolic pathways of complex diseases, helping to identify high-risk factors and potential biomarkers [110]. Moreover, peripheral neuropathy (PN) is a common complication in prediabetes and T2D. Multiple clinical studies have shown that obesity and dyslipidemia contribute to PN progression. Lipidomic studies have identified diacylglycerols as key subpathways for classifying obese individuals by PN status, with contributions from phosphatidylcholines, SMs, ceramides, and dihydroceramides [111]. Currently, Roux-en-Y gastric bypass (RYGB) surgery is an effective treatment for T2D and weight loss. Poss et al. found that RYGB led to a marked decrease in serum ceramide levels 2 years after surgery, and this decrease was sustained for up to 12 years, indicating that the alteration was not caused by postoperative stress. In addition, RYGB reduced the levels of nearly all ceramide species in patients with obesity, with the most significant effect on Cer(18:0) and Cer(22:0). Cer(18:0) is associated with IR; thus, its reduction may indicate improved glucose homeostasis. However, no study has yet found a link between Cer(22:0) and IR, making it valuable to investigate whether these ceramide species play a direct role in regulating glucose homeostasis. Furthermore, RYGB affects dihydroceramide levels, suggesting that it inhibits ceramide synthesis de novo [95]. Ceramide assays, an important component of lipidomics, have also been used to assess the effects of specific drugs on molecules in tissues. For example, fenofibrate treatment was found to reduce the plasma Cer(24:0)/Cer(16:0) ratio, with minimal effects on oxidative stress markers and no significant impact on inflammation [112].

Currently, first-line treatments for T2D include dietary modifications and metformin [110]. Given that ceramides are implicated in the development of IR, targeting ceramide metabolic pathways (such as SPT) presents a promising therapeutic approach for managing obesity and its associated disorders [3].

## 3. Challenges and Future Directions

Despite advances in understanding the role of ceramide in various disease processes, several limitations remain. Given the vast array of ceramide species, the precise physiological roles of individual ceramides are still incompletely understood [73]. For instance, understanding the specific functions of various ceramides in individuals with different skin types could lead to new applications in cosmetic and medical sciences by identifying species and concentrations relevant to skin health [113]. Applying dietary intervention as a viable strategy for lowering the concentration of circulating ceramides has been proposed in the current study. Such insights could enable the development of tailored dietary plans to enhance intervention outcomes and improve disease management [114].

Lipidomics, which employs both quantitative and qualitative assays, has benefited from advancements in assay technology, offering a more accurate evaluation of biomolecular properties. In experimental studies using pharmacological agents to alter lipid synthesis or metabolism, ceramide assays, an important part of lipidomics, can be used in conjunction with integrated genomic approaches, such as genomics, proteomics, metabolomics, and macrogenomics, to provide evidence of key cellular responses, including apoptosis, the cell cycle, and autophagy. These insights can further illuminate disease mechanisms [110]. Moreover, lipidomics has been applied to the study of neurodegenerative diseases and neuroinflammation, as well as the treatment of clinically complex metabolic disorders. When combined with artificial intelligence and interdisciplinary collaborations, lipidomics holds promise for advancing our understanding and treatment of these conditions [94].

## 4. Conclusions

Ceramide is a crucial component of sphingolipid metabolism, playing a significant role in cellular communication and constituting an essential biochemical molecule [6]. Along with their upstream and downstream metabolites, ceramides are involved in various pathological processes, including obesity, neurodegeneration, cancer, dermatitis, and CVD. Ceramide is a promising tool for diagnosis. The metabolic pathways of ceramides offer potential targets for controlling ceramide levels [3]. Thus, modulating enzymes linked to ceramide metabolism, introducing ceramide exogenously, or interfering with ceramide synthesis internally are valuable therapeutic strategies for alleviating the aforementioned conditions. Recent advancements in lipidomic technology have enabled the more accurate measurement of sphingolipid species in the brain, blood, and CSF [87]. Identifying specific sphingolipid species and their levels may enhance our understanding of the mechanisms underlying sphingolipid-mediated disorders, providing potential targets for future diagnostic, therapeutic and prognostic approaches. In addition, these advancements can facilitate further study of the effects of specific medications on tissue molecules. As mass spectrometry technology continues to advance and is applied in clinical practice, the ability to detect ceramide levels with greater precision is expected to yield new insights into disease prognosis and therapeutic treatments.

## Figures and Tables

**Table 1:** Analytical methods for ceramide detection.

Detective Method	Sample Matrix	Pretreatment Method	Chromatographic COLUMN	Mobile Phase	Gradient Elution	Linear Range	LOD (fmol) on Column
LC-ESI-MS/MS [17]	Serum	Liquid–liquid extraction	Luna C18column (150 mm × 2 mm ID, 5 µm particle size, 100 Å pore size)	Phase A: water: formic acid (100:0.1, *v*/*v*); Phase B: acetonitrile: tetrahydrofuran: formic acid (50:50:0.1, *v*/*v*/*v*)	0~0.6 min: A/B (60%/40%); 0.6~3.9 min: A/B (60%/40%~0/100%); 3.9~10.4 min: A/B (0/100%); 10.4~10.9 min: A/B (60%/40%).	Cer(18:0): 0.18–300 ng/mL; Cer(20:0): 0.24–400 ng/mL; Cer(24:1): 0.24–400 ng/mL; dhCer(24:0): 0.6–1000 ng/mL; dhCer(18:0): 0.3–500 ng/mL;	Not Mentioned
LC-ESI-MS/MS [18]	Plasma	Protein precipitation technology	Acquity BEH C18 (2.1 × 50 mm, 1.7 μm)	Phase A: 10 mM ammonium acetate + 0.1% formic acid in water; Phase B: 10 mM ammonium acetate + 0.1% formic acid in acetonitrile:2-propanol (4:3)	0~0.5 min: A/B (15%/85%); 0.5~1.5 min: A/B (15%/85%~0/100%); 1.5~4 min: A/B (0/100%); 4.5~5.0 min: A/B (15%/85%).	Cer(d18:1/16:0): 0.008~2 pmol/μL; Cer(d18:1/18:0): 0.008~2 pmol/μL; Cer(d18:1/24:0): 0.08~20 pmol/μL; Cer(d18:1/24:1): 0.08~20 pmol/μL.	Not Mentioned
LC-ESI-MS/MS [19]	Plasma	Protein precipitation technology	Water ACQUITY UPLC BEH Phenyl (3 mm × 100 mm, 1.7 nm)	Phase A: 0.2% formic acid, 1 mM ammonium formate aqueous solution Phase B: 0.2% formic acid, 1 mM ammonium formate methanol solution	0~2 min: A/B (20%/80%); 2~8 min: A/B (5%/95%); 8~9 min: A/B (2%/98%); 9~10 min: A/B (20%/80%).	Cer(d18:1/12:0): 1.953~8000 nM Cer(d18:1/14:0): 1.953~8000 nM Cer(d18:1/14:0): 1.953~8000 nM Cer(d18:1/18:0): 1.953~8000 nM Cer(d18:1/20:0): 1.953~8000 nM Cer(d18:1/22:0): 1.953~8000 nM Cer(d18:1/24:1): 1.953~8000 nM Cer(d18:1/24:0): 1.953~8000 nM	Not Mentioned
LC-APCI-MS/MS [20]	Fat Tissue	Liquid–liquid extraction and protein precipitation technology	Shimadzu Shim-pack C18 (50 mm × 2.1 mm, 2.0 µm)	Phase A: acetonitrile/water (6:4, ν/ν) + 0.1% formic acid + 0.1% ammonium formate; Phase B: acetonitrile/isopropanol (1:9, ν/ν) + 0.1% formic acid + 0.1% ammonium formate.	0–3 min: A/B (50%/50%); 4–12.5 min: A/B (30%/70%); 12.5–14 min: A/B (50%/50%).	Cer (d18:1/16:0): 125~5000 ng/mL Cer (d18:1/17:0): 1 µg/mL Cer (d18:1/18:0): 125~5000 ng/mL Cer (d18:1/20:0): 125~5000 ng/mL Cer (d18:1/24:0): 125~5000 ng/mL Cer [d18:1/24:1(15Z)]: 125~5000 ng/mL	Not Mentioned
LC-ESI-MS/MS [21]	Cell	Liquid–liquid extraction	ZORBAX Eclipse XDB-C8 (150.0 mm × 2.1 mm, 3.5 μm)	Phase A: 1 mmol/L ammonium formate and 0.2% formic acid in methanol solution, Phase B: 100% methanol	0~20 min: A/B (20%/80%~1/99%); 20~35 min: A/B (1%/99%~0/100%).	Cer16: 0.02~1.0 μg/mL; Cer17: 0.02~1.0 μg/mL; Cer24: 0.02~1.0 μg/mL; Cer18: 0.001~1.0 μg/mL.	Not Mentioned
LC-ESI-MS/MS [22]	Cosmetics	Ultrasound-assisted extraction	Waters XBridge Protein BEH C4 (2.5 μm, 2.1 mm × 50 mm)	Phase A: acetonitrile; Phase B: water.	0~2 min: A/B (40%/60%); 8~12 min: A/B (100/0%); 12.1~15 min: A/B (60%/40%).	Cer(t18:0/18:1): 5~100 ng/mL	Cer(t18:0/18:1): 0.125 µg/g
LC-APCI-MS/MS [23]	Fat Tissue	Liquid–liquid extraction	Shimadzu shim-pack GIST C18 (2.0 μm, 2.1 × 50 mm)	Phase A: acetonitrile/water [60:40, *v*/*v*] + 0.1%, formic acid + 0.1% formic acid, amine. Phase B: acetonitrile/iso-propanol [1:9, *v*/*v*] + 0.1% formic acid + 0.1% formic acid, amine.	0~1.0 min: A/B (50%/50%); 1.0~7.0 min: A/B (30%/80%); 7.1 ~8.5 min: A/B (50%/50%).	Not Mentioned	Not Mentioned
LC-ESI-MS/MS [24]	Tissues and Plasma	Liquid–liquid extraction	Zorbax SB-C8 (2.1 ×150 mm, 1.8 μm)	Phase A: 1 mM ammonium formate, 0.1% formic acid in water. Phase B: 2 mM ammonium formate, 0.1% formic acid in methanol.	0 min: A/B (80%/20%); 0–1.5 min: A/B (80%/20%~90%/10%); 1.5–2.3 min isocratic at A/B (90%/10%); 2.3–9.3 min: A/B (90%/10%~99/1%); 9.3–11 min isocratic at A/B (99/1%); 11–11.3 min:A/B (99%/1%~80%/20%); 11.3–13 min isocratic at A/B (80%/20%).	Not Mentioned	C14:0-Cer: 3.1 μg/mL. C16:0-Cer: 2.9 μg/mL C17:0-Cer: 2.8 μg/mL. C18:1-Cer:2.8 μg/mL. C18:0-Cer: 2.8 μg/mL. C20:0-Cer: 2.6 μg/mL. C24:1-Cer: 2.4 μg/mL. C24:0-Cer: 2.4 μg/mL.
LC-ESI-MS/MS [25]	Cells	Liquid–liquid extraction	RP C18 Nuecleosil AB column (5 μm, 70 × 2 mm I.D.)	Phase A: water-acetonitrilie-2-propanol (8:1:1, *v*/*v*/*v*); Phase B: acetonitrile-2-propanol (9:1, *v*/*v*)	0~2 min: A/B (35%/65%); 7~13 min: A/B (10%/90%); 15 min: A/B (0/100%); 16~18 min: A/B (35%/65%).	Not Mentioned	C16-Cer:0.253 ng/500 μg proteins. C18-Cer:0.253 ng/500 μg proteins. C20-Cer:0.253 ng/500 μg proteins

**Table 2:** (**a**) CERT1, CERT2, CERT2-TnT risk score. (**b**) CERT1, CERT2, CERT2-TnT risk rating.

**(a)**							
**Risk Score Table**	**Biomarkers**		**Score**				**Total Score**
			**Q1**	**Q2**	**Q3**	**Q4**	
CERT1	Cer(d18:1/16:0)		0	0	1	2	0~12
	Cer(d18:1/18:0)		0	0	1	2	
	Cer(d18:1/24:1)		0	0	1	2	
	Cer(d18:1/16:0)/Cer(d18:1/24:0)		0	0	1	2	
	Cer(d18:1/18:0)/Cer(d18:1/24:0)		0	0	1	2	
	Cer(d18:1/24:1)/Cer(d18:1/24:0)		0	0	1	2	
CERT2	Cer(d18:1/24:1)/Cer(d18:1/24:0)		0	1	2	3	0~12
	Cer(d18:1/16:0)/PC(16:0/22:5)		0	1	2	3	
	Cer(d18:1/16:0)/PC(14:0/22:6)		0	1	2	3	
	PC(16:0/16:0)		0	1	2	3	
CERT2-TnT	Cer(d18:1/24:1)/Cer(d18:1/24:0)		0	1	2	3	0~15
	Cer(d18:1/16:0)/PC(16:0/22:5)		0	1	2	3	
	Cer(d18:1/16:0)/PC(14:0/22:6)		0	1	2	3	
	PC(16:0/16:0)		0	1	2	3	
	hs-TnT		0	1	2	3	
**(b)**							
**Risk Score Table**	**Risk Rating**						
	**Low Risk**	**Medium Risk**	**Medium-high Risk**				**High Risk**
CERT1	0~2	3~6	7~9				10~12
CERT2	0~3	4~6	7~9				9~12
CERT2-TnT	0~4	5~7	8~10				11~15

**Table 3:** Ceramide levels in patients with cancer.

Study	Method	Participants		Sample	Conclusions	Future Perspectives
		Test	Control			
Grammatikos, 2016 [17]	LC-APCI-MS/MS	*n* = 122 HCC	*n* = 127 cirrhosis	Serum	The levels of long-chain and ultra-long-chain ceramides (C16–C24) were significantly higher in patients with HCC than in patients with cirrhosis (*p* < 0.001).	The C16 ceramide and its metabolite S1P might serve as new diagnostic markers of HCC in patients with liver disease.
Dubois N, 2016 [38]	LC-ESI-MS/MS	*n* = 35 colorectal CA before treatment	*n* = 35 colorectal CA after treatment	Plasma	Patients with controlled tumors within 1 year had higher ceramide levels, whereas 50% of patients with decreased ceramide levels experienced an increase in tumor volume.	Total plasma ceramide may serve as a biomarker of liver and lung oligometastases of colorectal cancer, enabling the classification of high-risk patients.
Kazuki Moro, 2017 [39]	LC-ESI-MS/MS	*n* = 44 breast CA	*n* = 36 peri-tumor and *n* = 44 normal breast tissues	Breast tissue	Cer(14:0), Cer(16:0), Cer(18:1), Cer(18:0), Cer(20:0), Cer(22:0), Cer(24:1), Cer(24:0), Cer(26:1), and Cer(26:0) were higher in breast cancer than in peritumor or normal breast tissue. Ceramide levels in cancer tissue were significantly negatively correlated with the nuclear grade (*p* = 0.04) and Ki-67 index (*p* = 0.09). The Area Under the Curve (AUC) scores of Receiver Operating Characteristic Curve (ROC) were 0.7226 for normal tissue and 0.7228 for peritumor tissue, respectively, showing that breast cancer tissue may be distinguished from normal breast tissue by ceramide levels.	Ceramide levels were higher in breast cancer tissue than in other tissues and were negatively correlated with aggressive phenotypes. Higher gene expressions of ceramide-related enzymes had a worse prognosis in breast cancer.
Jiang, 2018 [40]	LC-ESI-MS/MS	*n* = 15 hepatitis B related-AFP-negative HCC	*n* = 49 patients with hepatitis B cirrhosis	Serum	The expression level of Cer(d18:1/8:0)-1-P in patients with AFP-negative HCC was 2.177 nmol/mL, which was significantly higher than that in patients with hepatitis B cirrhosis (*p* < 0.05). The level of Cer(d18:1/8:0)-1-P can be used to identify hepatitis B-related AFP-negative HCC, with a sensitivity of 81.6% and a specificity of 86.7%.	Upregulated peripheral serum Cer(d18:1/8:0)-1-P might serve as a diagnostic marker of hepatitis B-related AFP-negative HCC.
Adam R. Markowski, 2020 [41]	LC-ESI-MS/MS	Colorectal tumor	Normal colorectal tissue	Colorectal tissue	The levels of sphingolipids in colorectal cancer tissues differed from those in surrounding healthy tissues, with increased levels of SPA, S1P, and Cer(14:0) and significantly lower levels of Cer(18:0) and Cer(20:0) in tumors. The levels of specific ceramides in colorectal cancer tissues and plasma depended on the stage of colorectal cancer. In ROC, sensitivities of plasma Cer(16:0), Cer(18:1), Cer(20:0), Cer(24:1) were 0.085, 0.092, 0.088, 0.088, respectively, in CRC(TNM III+IV).	Combined measurement of the plasma concentrations of several ceramides facilitates the differentiation between early and advanced lesions of colorectal cancer and is useful as a screening test for the detection of early colorectal cancer.
Xuewei Zhang, 2021 [42]	LC-ESI-MS/MS	CerS2-knockout cells	Control cells	Cell lipid	The CerS2-Cer(24:1) ceramide pathway limits ovarian cancer metastasis by restricting lamellipod formation in ovarian cancer cells. CerS2-Cer(24:1)	Provides insights for the development of ceramide-based therapies and the identification of biomarkers of metastatic ovarian cancer.

Abbreviation: HCC, hepatocellular carcinoma; CA, cancer; AFP, alpha fetoprotein; LS-MS/MS, liquid chromatography–tandem mass spectrometry; MS, mass spectrometry.

**Table 4:** Structures and nomenclature of ceramide in human SC.

	Fatty Acid	Non-Hydroxy Fatty Acid [N]	α-HYDROXY Fatty Acid [A]	β-HYDROXY Fatty Acid [B]	ω-Hydroxy Fatty Acid [O]	Esterified ω-Hydroxy Fatty Acid [EO]	Protein-Bound Fatty Acid [PO]
Sphingoid							
[DS]		CER[NDS]	CER[ADS]	CER[BDS]	CER[ODS]	CER[EODS]	CER[PODS]
[S]		CER[NS]	CER[AS]	CER[BS]	CER[OS]	CER[EOS]	CER[POS]
[P]		CER[NP]	CER[AP]	CER[BP]	CER[OP]	CER[EOP]	CER[POP]
[H]		CER[NH]	CER[AH]	CER[BH]	CER[OH]	CER[EOH]	CER[POH]
[SD]		CER[NSD]	CER[ASD]	CER[BSD]	CER[OSD]	CER[EOSD]	CER[POSD]

**Table 5:** Ceramide levels in patients with skin diseases.

Study	Method	Skin Diseases		Ceramides and Derivatives	Future Perspectives
		Test	Control		
Bo-Kyung Kim, 2021 [59]	LC-ESI-MS/MS	Psoriasis-like murine epidermis and human psoriatic stratum corneum	Healthy controls	Long-chain-ceramide ↓ Short-chain-ceramide ↑	IFN-γ may regulate ELOVL and CerS levels by downregulating transcription factors. Transcription factors, such as PPARs and liver X receptor agonists in the ceramide elongation process, may serve as potential therapeutic agents for lengthening the ceramide FA chain in psoriasis.
Fölster Holst, 2022 [60]	HPTLC	Ichthyoses lesional skin	Ichthyoses nonlesional skin	CER[EOS] ↓	Analysis of intercellular lipid lamellae organization and corneocyte membrane undulation may improve the understanding of the epidermal barrier in ichthyoses and assist in evaluating the effects of topical skin preparations.
Maria Rasmussen Rinnov, 2022 [61]	LS-ESI-MS/MS	Pediatric AD	Healthy controls	CER[DS] ↑ DHS ↑ CER[P] ↓	CER[P] had the highest prediction accuracy among the biomarkers (accuracy = 75.6%, sensitivity = 84%). The combination of five lipid ratios (CER[DS]-(d17:0)/CER[DS]-(d18:0), CER[DS]-(d18:0)/CER[S]-(d18:1), CER[P]- (t18:0)/CER[S]- (d18:1), CER[DS]- (d17:0)/CER[DS]- (d18:0), CER[S]- (d17:1)/CER[S]- (d18:1)) gave an accuracy of 89.4% to the prediction of AD within the first 12 months.
Dan Dai, 2022 [62]	LC-ESI-MS/MS	Patients with PVM	Patients with PV	Cer (d18:1/18:0) were positively correlated with the PASI in severe PV	Patients with PV at different severity levels have distinct metabolic profiles, aiding in understanding disease progression and establishing precision treatment strategies for PV.
Jihyun Kim, 2023 [63]	LC-ESI-MS/MS	AD lesional skin	AD nonlesional skin	C18-CER[NS] N-acylated with C16, C18, and C22 Fas ↑ C24-32 -CER [NS]/C14-22-CER [NS] and total CER[EO]/total CER[NS] were negatively correlated with transepidermal water loss.	Pediatric AD skin showed aberrant lipid profiles associated with microbial dysbiosis and cutaneous barrier dysfunction.
Evgeny Berdyshev, 2023 [64]	LC-ESI-MS/MS	Children with AD family history	Children without AD family history	CER[PO] ↓ Short-chain CER[N] and CER[A] ↑	Noninvasive skin tape strip analysis of ceramides can identify asymptomatic children at risk of future AD with high probability. A combination of lipids and cytokines serves as a powerful biomarker for predicting AD development, paving the way for precision medicine in AD.
Mateusz Matwiejuk, 2023 [65]	LC-ESI-MS/MS	Patients with psoriasis	Healthy controls	Positive associations between CER_t and CER_s, SFA_t and CER_s, and SFO_t and CER_s	Sphingolipid metabolism is impaired in both the affected skin and serum in patients with psoriasis. Skin and serum lipids show interrelationship, suggesting systemic involvement and correlations between specific sphingolipids.
Howard Chu, 2023 [66]	LC-ESI-MS/MS	AD with HND	AD without HND	CER[EOS] ↓ CER[EOP] ↓	
Qianqian Su, 2024 [67]	LC-ESI-MS/MS	Acne in women	Healthy controls	Ceramide chain length ↓	Skin surface lipids are closely associated with acne development. Lipidomics is a useful tool for analyzing skin surface lipids in different types of acne.

Symbol: ↑ means increase; ↓ means decrease. Abbreviation: AD, atopic dermatitis; DHS, dihydrosphingosine; CER[P], phytosphingosine; C18 -CER [NS], ceramide with C18 sphingosine as its sphingoid base and nonhydroxy fatty acids; SM, sphingomyelin; LPC, lysophosphatidylcholine; SFO, sphingosine; S1P, Sphingosine, sphingosine-1-phosphate; SFA, saturated fatty acids; CER_t, ceramide in tissue; CER_s, ceramide in serum; SFA_t, sphinganine in tissue; SFO_t, sphingosine in tissue; ELOVLs, elongases of very-long-chain fatty acids; PV, psoriasis vulgaris; PVM, psoriasis vulgaris with metabolic diseases; PC, phosphatidylcholine; PASI, Psoriasis Area and Severity Index; HND, head and neck dermatitis; FAs, fatty acids; TG, triglycerides; PI, phosphatidylinositol; HPTLC, high-performance thin layer chromatography; high-throughput UPLC-QTOF-MS, high-throughput ultra-performance liquid chromatography–quadrupole time-of-flight mass spectrometry.

**Table 6:** Three stages of AD based on its main signs and symptoms.

Stage	Characteristics/Symptoms
Preclinical AD	1. Measurable biomarkers and detectable changes in the brain, CSF, and blood.
	2. Absence of symptoms such as memory loss.
MCI due to AD	1. Measurable biomarkers and detectable changes in the brain related to AD pathology
	2. Moderate cognitive decline, mainly affecting the performance of small daily tasks (such as paying bills or preparing meals).
Dementia	1. Measurable biomarkers and detectable changes in the brain, related to AD pathology.
	2. Substantial memory loss.
	3. Behavioral and personality changes.
	4. Severe impairments in completing daily tasks.

**Table 7:** Alterations in ceramide and its metabolites as well as in other lipids in AD.

Study	Method	Skin Diseases/Condition		Sample	Ceramides and Derivatives	Future Perspectives
		Test	Control			
Michelle M. Mielke, 2010 [78]	LC-ESI-MS/MS	Patients with MCI	Healthy controls	Plasma	Cer(22:0) ↓ Cer(24:0) ↓	Ultra-long-chain ceramides in the plasma predict memory loss and right hippocampal volume loss in patients with MCI and may be early indicators of AD progression.
PeñaBautista Carmen, 2022 [76]	LC-ESI-MS/MS	Preclinical AD	Healthy controls	Plasma	Cer↑	The study of lipid profiles in plasma samples can help identify early stages of AD and potential new biomarkers.
		MCI-AD	Healthy controls			
Daan van Kruining, 2023 [73]	LC-ESI-MS/MS	Men with MCI	Healthy controls	Plasma	Cer(18:0) ↑ Cer(24:1) ↑ Ceramide chain lengths ↑ (Cer(20:0), Cer(22:0), and Cer(24:1) ↑ are associated with larger volume of the hippocampus)	The study highlights the importance of considering sex and age-related factors when examining sphingolipid and CERT metabolism related to cognitive function. No associations of plasma sphingolipids with MCI or brain volumes were found in women. Further analyses of plasma ceramides as potential markers of MCI in middle-aged men are warranted.

Symbol: ↑ means increase; ↓ means decrease. Abbreviation: MCI, mild cognitive impairment; AD, Alzheimer’s disease; LPE, lysophosphatidylethanolamines; MG, monoglycerides; DG, diglycerols; LPC, lysophosphatidylcholine; PE, phosphatidylethanolamines; PI, phosphatidylinositols; UPLC-TOF/MS-Orbitrap QExactive Plus MS, ultra-performance liquid chromatography coupled to time-of-flight mass spectrometry. AD is closely associated with comorbidities such as diabetes, hypertension, depression, and sleep disorders. Obstructive sleep apnea (OSA) is highly prevalent among patients with AD, and approximately 40% of patients with AD have severe OSA. Both AD and OSA have been linked to the dysregulation of lipid metabolism. CSF ceramide assays have revealed a lipidomic fingerprint that can identify patients with AD and severe OSA [79]. In addition, numerous studies have demonstrated that AD is a crucial comorbidity in patients with type 2 diabetes (T2D), with patients with T2D having a 1.4- to 2-fold increased risk of AD. Mechanistic insights from animal models and in vitro experiments suggest that lipotoxicity contributes to neurological dysfunction and neurodegeneration. The pathway of lipotoxicity-induced injury, which involves alterations in ceramide synthesis, may be a common molecular mechanism in the pathogenesis of both T2D and AD [80].

**Table 8:** Alterations in ceramide levels in various neurodegenerative diseases.

Study	Method	Nervous System Diseases/Condition		Sample	Ceramides and Derivatives	Future Perspectives
		Test	Controls			
Xing Y, 2016 [83]	LC-ESI-MS/MS	PDD	PD-NC	Plasma	Cer(14:0) ↑ Cer(24:1) ↑ Cer(22:0), Cer(20:0), and Cer(18:0) were associated with hallucinations, anxiety and sleep behavior disturbances, respectively	In PDD, increased ceramide levels were correlated with decreased memory function and a higher odd of multiple neuropsychiatric symptoms.
Emer R McGrath, 2020 [84]	LC-ESI-MS/MS	Dementia-free Framingham Offspring Study cohort		Plasma	Cer22:0/Cer16:0 ↓ Cer24:0/Cer16:0 ↓	Circulating ceramide ratios may serve as biomarkers for predicting dementia risk in cognitively healthy adults.
Hideki Oizumi, 2022 [85]	LC-ESI-MS/MS	ND groups (including IPD, DLB, MSA, AD, and PSP)	Healthy controls	Plasma	S1P ↓	The study indicates the important role of abnormal sphingolipid metabolism in neurodegeneration.
Lv Hong, 2022 [86]	LC-ESI-MS/MS	Patients with PSD	Patients without PSD	Plasma	Cer(16:0) ↑ Cer(18:0) ↑ Cer(24:0) ↑ Cer(24:1) ↑	Serum ceramides may become an essential candidate biomarker for PSD diagnosis and may aid in monitoring the other biomarkers in the pathway.
		Patients with PSD	Patients with MD		Cer(16:0) ↑ Cer(18:0) ↑ Cer(24:0) ↑	
Koushik Mondal, 2024 [87]	LC-ESI-MS/MS	TBI mouse model	Healthy controls	Brain tissue	Sphingosine ↑ C1P ↑	Alterations in sphingolipid metabolite composition, particularly sphingomyelinases and short chain ceramides, may contribute to the induction and regulation of neuroinflammatory events in the early stages of TBI, suggesting targets for novel diagnostic, prognostic, and therapeutic strategies in the future.
				Plasma	Cer(22:0)) ↓ (at 1 day) Cer (22:0) ↑ (at 7 days) Cer (24:1) ↑ (at 3 days) Cer (24:1) ↓ (at 7 days) Cer (24:0) ↓ SM (22:0) ↓ (at 3 days) SM (22:0) ↑ (at 7 days)	

Symbol: ↑ means increase; ↓ means decrease. Abbreviation: CERT, ceramide transfer protein; PD, Parkinson’s disease; ND, neurodegenerative disease; IPD, idiopathic Parkinson’s disease; DLB, dementia with Lewy bodies; MSA, multiple system atrophy; AD, Alzheimer’s disease; PSP, progressive supranuclear palsy; S1P, sphingosine-1-phosphate; TBI, traumatic brain injury; C1P, ceramide-1-phosphate; SM, sphingomyelins; MHC, monohexosylceramide; PDD, Parkinson’s disease dementia; PD-NC, Parkinson’s disease with no cognitive impairment; MD, major depression; PSD, poststroke depression.

**Table 9:** Alterations in ceramide levels in patients with obesity.

Study	Method	Participants		Sample	Main Findings	Conclusions
		Test	Controls			
Haus Jacob, 2009 [101]	LC-ESI-MS/MS	*n* = 13 T2D	*n* = 14 healthy control	Plasma	Patients with T2D had higher levels of Cer(d18:1/18:0), Cer(d18:1/20:0), Cer(d18:1/24:1), and total ceramides. Insulin sensitivity was inversely correlated with Cer(d18:1/18:0), Cer(d18:1/20:0), Cer(d18:1/24:1), Cer(d18:1/24:0), and total ceramides.	Plasma ceramide levels were increased in patients with obesity and T2D and were positively correlated with insulin resistance.
Ximena Lopez, 2013 [102]	LC-ESI-MS/MS	*n* = 14 women with obesity and T2D	*n* = 14 women with obesity and T2D	Fasting plasma	Cer(d18:1/22:0) and Cer(d18:1/20:0) were elevated, and Cer(d18:1/18:0) and Cer(d18:0/24:1) were twice that in healthy individuals (*p* < 0.05).	Plasma ceramides were elevated in T2D, reflecting tissue insulin resistance, potentially due to low adiponectin levels.
Jeremy Warshauer, 2015 [103]	LC-ESI-MS/MS	*n* = 19 pioglitazone	*n* = 18 placebo	Plasma	Cer(d18:1/18:0), Cer(d18:1/20:0), Cer(d18:1/24:1), Cer(d18:0/18:0), Cer(d18:0/24:1), lactosylceramides Cer(d18:1/16:0), hexosylceramides Cer(d18:1/16:0), Cer(d18:1/16:0), and Cer(d18:1/22:0) were markedly reduced after 6 months of pioglitazone treatment (all *p* < 0.01).	Plasma ceramide levels were markedly decreased in patients with MetS who received pioglitazone for 6 months. Some changes were correlated with insulin resistance and adiponectin levels.
Jacob Haus, 2017 [104]	LC-ESI-MS/MS	*n* = 76 SS	*n* = 76 IMF	Skeletal muscle lipids	SS ceramides, especially those whose chain length was C16 to C18 (Cer(d18:1/16:0) and Cer(d18:1/18:1)) or generated under the stimulation of plasma palmitate, were associated with biomarkers of insulin resistance. However, the IMF level was not correlated with any metabolic parameters.	Skeletal muscle SS ceramides, especially C16~18 chain lengths, and the de novo synthesis of intramyocellular ceramide from plasma palmitate are associated with insulin resistance markers.
Hady Razak Hady, 2019 [105]	LC-ESI-MS/MS	*n* = 31 IGT group (women = 12, men = 19), and *n* = 33 T2D (women = 19, men = 14)	Normal glucose tolerance group (NGT, women = 30, men = 36)	Liver	Hepatic ceramides were higher in women with T2D than in women with NGT (*p* < 0.05). Glycemic parameters, such as FBG, OGTT at 120 min, and HbA1c, were correlated with ceramides. Hepatic ceramides were higher in men with IGT than in men with NGT, but only Cer(d18:1/22:0) was correlated with all glycemic parameters	Ceramide contributed to the induction of hepatic insulin resistance, and it may differ between men and women.
Luis Felipe León-Aguilar, 2019 [106]	LC-ESI-MS/MS	*n* = 91 docosahexaenoic acid	*n* = 92 placebo	Plasma	The total abundance of plasma ceramides in overweight and obese mothers, especially Cer(d18:1/20:0), Cer(d18:1/22:0), Cer(d18:1/23:0), and Cer(d18:1/24:0), was significantly decreased. Compared with children of normal weight mothers, the levels of Cer(d18:1/22:0), Cer(d18:1/23:0), and Cer(d18:1/24:0) were similar in the 4-year-old children of overweight or obese mothers.	Maternal obesity led to long-term changes in plasma ceramide levels of their offspring, and lipids may serve as early predictors of metabolic disease risk that result from maternal obesity.
Yuan, 2021 [107]	LC-APCI-MS/MS	*n* = 56 OD	*n* = 144 OND	Abdominal adipose tissue	Cer(d18:1/16:0), Cer(d18:1/18:0), Cer(d18:1/24:0), Cer(d18:1/24:0), and total ceramides in the fat tissue of OD group were higher than those in the OND group (*p* < 0.05), whereas the difference in Cer(d18:1/20:0) was not statistically significant (*p* > 0.05). IL-1 and IL-18 in serum and fat tissue of OD group were higher than those in the OND group (*p* < 0.05).	Ceramide level in the fat tissue of patients with obesity was associated with the inflammation of fat tissue and increased diabetes risk.
Jakub Morze, 2022 [108]	Meta-analysis	*n* = 11,771 T2D	*n* = 59,425 healthy controls	Plasma, serum, and urine	Higher plasma and serum levels of phosphatidylethanolamines and ceramides included in the meta-analysis were associated with a higher risk of type 2 diabetes.	Several plasma and serum metabolites, including amino acids, lipids, and carbohydrates, were associated with the risk of type 2 diabetes.
Kelli Lytle, 2023 [109]	LC-ESI-MS/MS	*n* = 25 obesity		Liver, plasma, and VLDL particles	(i) The proportion of Cer(14:0), Cer(18:0), Cer(20:0), and Cer(24:1) in the liver and whole plasma were positively correlated. (ii) Hepatic fat was positively correlated with the proportion of hepatic Cer18:1, Cer18:0, and Cer20:0 but not with total hepatic ceramide concentration. (iii) The proportions of whole plasma ceramide subspecies, especially Cer(14:0), Cer(18:0), Cer(20:0), and C(24:1) chain length, are reflective of those of hepatic ceramide subspecies in individuals with obesity.	A correlation was observed between the levels of ceramides in the liver and plasma of patients with obesity.

Abbreviations: T2D, type 2 diabetes; SS, subsarcolemmal; IMF, intramyofibrillar; IGT, impaired glucosetolerance; OD, obesity with diabetes; OND, obesity without diabetes; VLDL, low-density lipoproteins; QTMS/MS, quantitative tandem mass spectrometry.

## Data Availability

No datasets were generated or analyzed during the current study.
